# Emergency Department Management of Acute Heatstroke: A Retrospective Analysis from Phoenix, Arizona

**DOI:** 10.5811/westjem.42051

**Published:** 2025-09-25

**Authors:** Jeffrey R. Stowell, Paul Pugsley, Megan McElhinny, Geoffrey Comp, Jacquelyn Pearlmutter, Murtaza Akhter, David Sklar

**Affiliations:** * Creighton University School of Medicine, Phoenix, Department of Emergency Medicine, Phoenix, Arizona; † University of Arizona College of Medicine, Phoenix, Department of Emergency Medicine, Phoenix, Arizona; ‡ Valleywise Health, Department of Emergency Medicine, Phoenix, Arizona; § Penn State Health Milton S. Hershey Medical Center, Department of Emergency Medicine, Hershey, Pennsylvania; || HCA Healthcare, Department of Emergency Medicine, Miami, Florida; # Arizona State University, School of Medicine and Advanced Medical Engineering, Tempe, Arizona

## Abstract

**Introduction:**

The global incidence and severity of severe heat illness is on the rise. The increasing number of summer heatwaves in Phoenix, Arizona, gave us a distinctive opportunity to better understand the impact on the clinical presentation and management of acute heatstroke. Our primary objective in this study was to describe the prehospital and emergency department (ED) clinical presentation, treatment, and outcomes of patients with acute heatstroke at a single hospital system during the summers of 2021 and 2022 in Phoenix.

**Methods:**

This was a descriptive, retrospective observational study of heatstroke-associated adult ED presentations occurring from June 1 – August 31, 2021 and June 1 – August 31, 2022, to a single hospital system in Maricopa County.

**Results:**

We identified 60 ED heatstroke encounters. The median environmental daily maximum (Tmax) and minimum (Tmin) were 106.0° Fahrenheit (interquartile range [IQR]) 102.0 – 109.0°F) and 84.0°F (IQR 79.0 – 88.0°F), respectively. The patients were commonly male (42, 70.0%, 95% CI 56.8 – 81.2%), White (26, 43.3%, 95% CI 30.6 – 56.8%), middle-aged (mean 52.7 years, 95% CI 48.4 – 56.9), Medicaid-insured (37, 61.7%, 95% CI 48.2 – 73.9%), and presenting via emergency medical services (60, 100%). Patients were commonly of high acuity (median Emergency Severity Index 1, IQR 1.0 – 2.0), and intubated (45, 75.0%, 95% CI 62.1–85.3%). Forty-seven (78.3%, 95% CI 65.8 – 87.9%) patients were found unresponsive outside with associated substance use (methamphetamines 22, 46.8%, 95% CI 32.1 – 61.9%; and fentanyl 14, 29.8%, 95% CI 17.3 – 44.9%). The average patient Tmax at ED presentation was 41.9°C (IQR 41.1 – 42.2). Forty-one patients (68.3%, 95% CI 55.0 – 79.7%) survived to hospital discharge or transfer, of whom 32 (82.1%, 95% CI 66.5 – 92.5%) were neurologically intact.

**Conclusion:**

During the summers of 2021 and 2022, a significant number of heatstroke presentations were treated in a single healthcare system in Maricopa County, Arizona. A substantial number were successfully treated with cold water immersion and discharged neurologically intact. In this urban population, extreme weather exposure and associated substance use appeared to play significant roles.

## INTRODUCTION

Climate change has contributed to an increase in global heatwave frequency and severity, which is predicted to continue for decades to come.^1^–^3^ As a result, the global incidence of heat illness is on the rise.^3^ Severe heat-associated illness, such as heatstroke, is a significant cause of morbidity worldwide. Heatstroke mortality rates approach 50% in its most severe forms and disproportionately impacts the elderly and very young, as well as individuals with comorbidities, economic stress, and social disadvantages.^2^,^4^–^9^ Climate change modeling predicts the number of excess deaths due to heat illness to increase 233% by midcentury.^10^

In Phoenix, Arizona, the Maricopa County Department of Public Health (MCDPH) recorded an increase in heatstroke deaths from 61 in 2014 to 425 in 2022.^11^,^12^ In 2021 and 2022, the city of Phoenix experienced its 10^th^ and 12^th^ hottest years in recorded history, respectively.^13^ During the 2021 and 2022 summers, record maximum temperatures reached as high as 118°F, with 22 days of temperatures ≥ 110°F (43.3°C) in each of those years.^13^ Our primary objective in this study was to describe the prehospital and emergency department (ED) presentation of severe heat-associated illness, including the causes, clinical features, therapeutic approaches, and outcomes at a single hospital system during the summers of 2021 and 2022. A description of these patients will help expand our current understanding of the presentation and management of heat-associated illness, assist in developing additional preventative and therapeutic opportunities, and better prepare emergency physicians and public health professionals for the future impact of extreme heat events.

## METHODS

### Study Design

We conducted a retrospective, observational study of heat-associated, individual adult ED visits over two consecutive summers between June 1–August 31, 2021 and June 1–August 31, 2022, to a single hospital system located in the Phoenix, Arizona, metropolitan area of Maricopa County. The study protocol was approved by the local institutional review board. No author conflicts of interest were identified prior to data collection.

### Study Population and Setting

The study was conducted across three EDs in a large, urban, multisite, safety-net healthcare system. The annual combined ED census of the hospital system is ≈ 107,000 total patient encounters. The ED uses a variety of approaches to patient cooling including non-immersion cooling (eg, the application of cold towels, ice packs, fans, etc) and cold water immersion.^14^ The selection and deployment of cooling therapy is at the discretion of the treating emergency medicine physician.

Located in the Sonoran Desert, Maricopa County has a population of ≈ 4.551 million people living in a catchment area of 9,224 square miles.^15^ The arid climate is traditionally hottest from May – October with relatively low humidity.^11^ Annually during 2006 – 2016, the county had a mean of 140 days with daily maximum temperatures of ≥ 90 °F (32 °C) and a mean of 57 days with daily maximum temperatures ≥ 105 °F (40.5 °C).^16^ The Arizona Emergency Information Network defines “extreme heat” as at least 2–3 days with temperatures above 90 °F.^17^ In 2022, 424 heat-associated deaths were identified in Maricopa County, 58% of which occurred in July^12^; this was a 25% increase over 2021. A 2005 heat event in Maricopa County prompted the formation of the City of Phoenix Heat Relief Network.^11^ Cooling stations in Maricopa County were open and available to the public throughout the study time frame.^18^

**Population Health Research Capsule**

What do we already know about this issue?

*The incidence and severity of global heatwaves and heat illness are rising. Heatstroke is a significant cause of morbidity with mortality rates approaching 50%*.

What was the research question?

*We sought to describe the presentation of heatstroke, including the causes, clinical features, therapeutic approaches, and outcomes*.

What was the major finding of the study?

*68.3% (CI 55.0 – 79.7%) of heatstroke victims survived to hospital discharge, with 82.1% (CI 66.5 – 92.5%) neurologically intact*.

How does this improve population health?

*A description of how severe heat-associated illness presents to the ED helps expand our understanding of its presentation and management and develop preventative measures*.

### Study Protocol

Adult patients ≥ 18 years of age who presented to the ED with heatstroke from June 1 – August 31, 2021, and June 1 – August 31, 2022, were included in the study. We defined heatstroke as the combination of a temperature of ≥ 40.0 °C and central nervous system dysfunction (eg, altered level of consciousness) during the prehospital or ED course, which was either attributable to or associated with environmental heat exposure.^9^,^19^ Heatstroke was further defined as “classic” if due to the inability to avoid or physiologically adjust to heat exposure (eg, elderly or chronically ill persons), or “exertional” if associated with strenuous physical activity (eg, outdoor labor or activity).^19^ Encounters that could not be defined as either were classified as “unclassified.” We defined ED cooling as the time from the first ED core temperature recorded to the first temperature of ≤ 39 °C. We excluded from analysis pediatric patients < 18 years of age at the time of ED presentation, and those with presentations that could not be attributed to heatstroke after chart review.

We collected participant demographics, prehospital, ED, and hospitalization course information through chart extraction. We used multiple chart review and retrospective study best practices, including the Strengthening the Reporting of Observational Studies in Epidemiology guidelines, during study design, which included the creation of a standardized abstraction codebook^20^–^23^ (Supplemental Table 1). Three of the study investigators—experienced emergeny medicine physicians— extracted study data from the electronic health record (Epic Systems Corporation, Verona, WI). Prior to data collection they were trained per the study protocol, with ongoing education and review as needed. Training included an in-person guided review session with each extractor and supervised extraction of initial data. They used the codebook when extracting data from ED encounters that met study inclusion criteria. Discrepancy or disagreement was adjudicated through cross-abstractor validation.

### Study Measurements

Demographics included patient age, gender, race, primary language, financial class, housing status, and primary heat illness diagnosis. The prehospital course included the following: pick-up location (ZIP code); initial non-core temperature; documentation of cardiopulmonary arrest; administration of naloxone and prehospital cooling; airway management; and narrative information regarding the circumstances of the heat exposure. The ED course included ED arrival method (private vehicle vs emergency medical services [EMS]), Emergency Severity Index (ESI) score, maximum core temperature (Tmax), Glasgow Coma Scale (GCS) score, ED intubation, urine drug screen (UDS) results, ethanol level, whether cardiopulmonary arrest occurred in the ED, ED cooling methods, and ED disposition (admit, discharge, death). Hospital course included admission level of care (intensive care unit [ICU], step-down, ward), hospital disposition (discharge or death), and neurologic status at the time of hospital disposition (per physician documentation, return to baseline neurologic status, new disabled, disposition needs, etc).

Investigators also collected publicly available Maricopa County 2021 and 2022 meteorologic, heat surveillance, and heat relief resources. Meteorologic data from June 1 – August 31, 2021, and June 1 – August 31, 2022, included maximum and minimum daily environmental temperatures, and heat advisory dates.^24^,^25^ The type of heat relief resources including locations of cooling center was obtained from the Office of Heat Response and Mitigation.^26^

### Study Analysis

This study was designed as a descriptive analysis of ED heatstroke presentations that occurred in the summers of 2021 and 2022. We identified and described presentations of acute heatstroke, including patient demographics, diagnostics, and therapeutic cooling interventions. Confidence intervals for frequencies were calculated using the Aggresti-Couli method (Wald approximation with correction). We made group comparisons using means and the Student *t*-test for normally distributed data, and medians and Mann-Whitney U testing for non-normally distributed data. Additionally, we described Phoenix, Arizona, meteorologic data using mean, median, interquartile range, and CIs, where appropriate. A graphical representation of heatstroke presentations geolocated to heat relief resources was created using Google Maps (Google LLC, Mountain View, CA). If study participant data were incomplete or unavailable, those encounters were noted in the results as “unknown.” We performed statistical analysis using Microsoft Excel (Microsoft Corp, Redmond, WA) and R v4.0.5 (The R Foundation for Statistical Computing, Vienna, Austria).

## RESULTS

**Figure 1:**
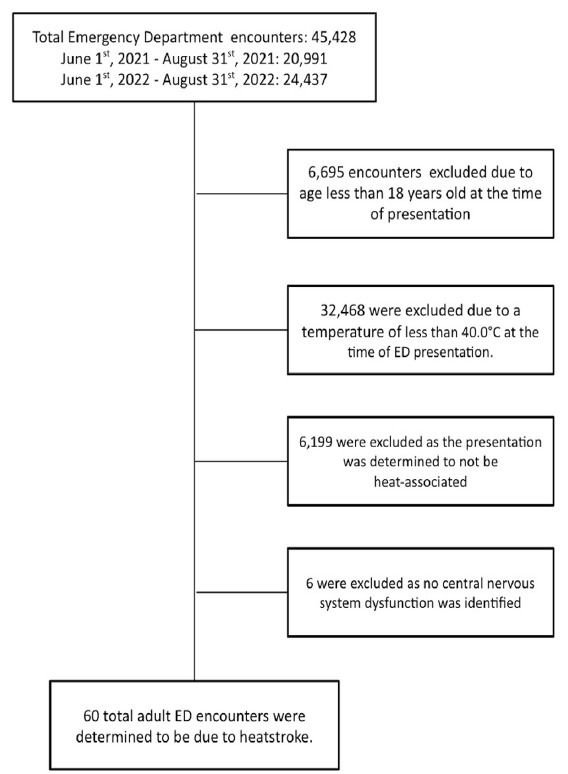
Total number of emergency department encounters and reasons for exclusion from heatstroke study. *ED*, emergency department

From June 1 – August 31, 2021, and June 1 – August 31, 2022, 45,428 total ED encounters occurred at the study site. Of these, we excluded 6,695 patients < 18 years of age at the time of ED presentation. Of the adult encounters, 32,468 were excluded due to a temperature < 40.0 °C. Of those patients with a temperature of ≥ 40.0 °C at the time of ED presentation, 6,199 were excluded because the presentation was unrelated to environmental heat exposure (eg, infection). Of the remaining encounters, we excluded an additional six patient encounters that were determined not to meet the definition of heatstroke as no central nervous system dysfunction was identified during either the prehospital or ED course (eg, heat exhaustion). A total of 60 adult ED encounters from June 1 – August 31, 2021, and June 1 – August 31, 2022, were determined to be due to acute heatstroke (Figure 1).

Heatstroke patients were most commonly male (42, 70.0%, 95% CI 56.8 – 81.2%), White (26, 43.3%, 95% CI 30.6 – 56.8%), middle-aged (mean 52.7 years, 95% CI 48.4 – 56.9%), Medicaid-insured (37, 61.7%, 95% CI 48.2 – 73.9%), presenting via EMS (60, 100%), of high acuity (median Emergency Severity Index [ESI] – 1, IQR 1.0 – 2.0), having associated substance use (32, 53.3%, 95% CI 40.0 – 66.3%), and being homeless (22, 36.7%, 95% CI 24.6 – 50.1%). During the study period, the general ED population was 49.6% male, 78.4% White, 44.9% Medicaid-insured, and arrived via EMS in 22.8% of encounters. In the study population, 26 (43.3%, 95% CI 30.6 – 56.8%), and 16 (26.7%, 95% CI 16.1 – 39.7%) patients’ UDS results were positive for methamphetamines and fentanyl, respectively, while 14 (23.3%, 95% CI 13.4 – 36.0%) were positive for both. Twenty-three patients (38.3%, 95% CI 26.1 – 51.8%) received naloxone prior to ED arrival; 45 (75.0%, 95% CI 62.1 – 85.3%) were intubated, three (5.0%, 95% CI 1.0 – 13.9%) in the prehospital setting. Nineteen (31.7%, 95% CI 20.3 – 45.0%) of the heatstroke patients did not survive to hospital discharge, with 15 (25.0%, 95% CI 14.7– 37.9%) and four (6.7%, 95% CI 1.8 – 16.2%) dying in the ED and inpatient setting, respectively. Of 14 patients (23.3%, 95% CI 13.4 – 36.0%) who suffered prehospital cardiopulmonary arrest, none were successfully resuscitated in the ED and they contributed to all but one of the ED deaths. Of the 60 patients with heatstroke, 45 survived beyond the ED, with seven (11.7%, 95% CI 4.8 – 22.6%) discharged from the ED and 38 (63.3%, 95% CI 49.9 – 75.4%) admitted to the hospital. Of those admitted, 31 (81.6%, 95% CI 65.7 – 92.3%) were admitted to the ICU. A total of 41of the 60 patients (68.3%, 95% CI 55.0 – 79.7%) survived to hospital discharge or transfer. Of the 39 who were discharged, 32 (82.1%, 95% CI 66.5 – 92.5%) were neurologically intact at the time of discharge. Study participant demographics are in Table 1.

The median patient, prehospital non-core Tmax was 41.1°C (IQR 40.6 – 42.0 °C); core Tmax at ED presentation was 41.9°C (IQR 41.1 – 42.2) and post-cooling minimum temperature (Tmin) was 37.0°C (IQR 35.9 – 37.7°C). Thirty-six (60.0%, 95% CI 46.5 – 72.4%) patients had documentation of EMS-initiated prehospital cooling interventions. Prehospital cooling techniques included the use of or combination of cold intravenous fluids (22 patients), placement of ice packs (16), placement of wet towels (3), cold blanket (1), and other “unspecified” cooling measures (11). The ED cooling techniques included cold water immersion in 38 (63.3%, 95% CI 49.9 – 75.4%), non-immersion cooling (eg, cold towels, ice packs, fans, etc) in 13 (21.7%, 95% CI 12.1 – 34.2%), and no cooling in 9 (15.0%, 95% CI 7.1 – 26.6%) of the heatstroke patients.

Patients treated with cold water immersion therapy demonstrated an overall higher acuity with higher median ESI (1.0 IQR 1.0–2.0 vs. 2.0 IQR 1.0 – 2.0), median Tmax (42.0°C, 95% CI 41.3 – 42.3°C vs. 40.7°C, 95% CI 40.2 – 41.6°C), intubation rate (78.9%, 95% CI 62.7 – 90.5% vs 68.2%, 95% CI 45.1 – 86.1%), rate of prehospital cardiac arrest (15.8%, 95% CI 6.0 – 31.3% vs. 7.7%, 95% CI 0.2 – 36.0%), and lower median Glascow Come Scale (3 (IQR 3.0 – 6.0) vs. 11 (IQR 7.0–13.0)) as compared to non-immersion cooling. In patients where the duration of cooling was documented, the median temperature reduction, duration, and rate of cooling was 3.06°C (95% CI 2.4 – 3.6°C), 35.0 minutes (IQR 26.3 – 56.3), and 0.08°C per minute (95% CI 0.05 – 0.12 °C) for cold water immersion therapy (34, 89.5% immersion encounters with documentation), and 2.65°C (95% CI 2.18 – 3.10 °C), 60.5 minutes (IQR 37.5 – 87.3), and 0.04 °C per minute (95% CI 0.03–0.06°C) for non-immersion (12, 92.4%). Cold Water Immersion study participant demographics and outcomes presented in Table 2.

The overall mortality rates were 28.9% (95% CI 15.4 – 45.9%) for cold water immersion cooling and 7.7% (95% CI 0.2 – 36.0%) for non-immersion cooling. The “no-cooling” patients demonstrated the highest overall prehospital acuity. Seven of the nine no-cooling patients presented to the ED in cardiopulmonary arrest, likely contributing to the decision to pursue immediate Advanced Cardiac Life Support (ACLS) measures rather than initiation of ED cooling.

Only nine (15.0%, 95% CI 7.1 – 26.6%) of the heatstroke presentations were attributable to a “classic” cause of heat exposure. Forty-seven (78.3%, 95% CI 65.8 – 87.9%) patients were found down outside and were difficult to classify distinctively as either classic or exertional heatstroke. These encounters were classified as “unclassified” causes of heatstroke. Further, four (6.7%, 95% CI 1.8 – 16.2%) patients expired without sufficient clinical context to determine the cause of the heat exposure. The “classic” heatstroke encounters were 44.4% male (95% CI 13.7 – 78.8%), median 62.0 years old (IQR 56.0–72.0), 100% domiciled (95% CI 66.4 – 100.0%), 55.6% intubated (95% CI 21.2 – 86.3%), and 88.9% (95% CI 51.8–99.7%) survived to hospital discharge or transfer. Two (22.2%, 95% CI 2.8 – 60.0%) of these presentations had associated methamphetamine use. The “unclassified” heatstroke presentations were predominately male (36, 76.6%, 95% CI 62.0 – 87.7%), younger (49.8 years old, 95% CI 45.3 – 54.3), homeless (22, 46.8%, 95% CI 32.1 – 61.9%), and associated with substance use (methamphetamines 22, 46.8%, 95% CI 32.1 – 61.9%, and fentanyl 14, 29.8%, 95% CI 17.3 – 44.9%). A description of the 47 “unclassified” heatstroke presentations is available in Table 3.

**Figure 2:**
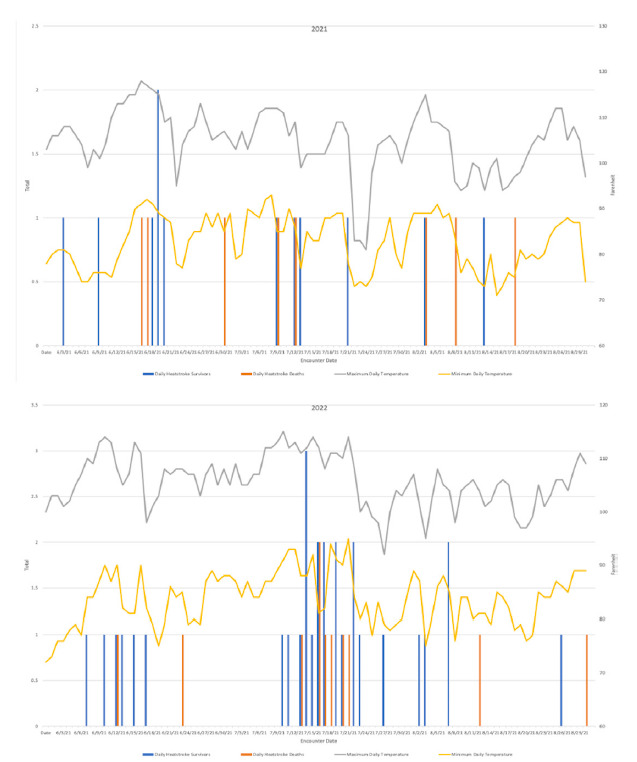
2021 and 2022 heatstroke and heat-related death as compared to environmental temperatures.

The median environmental daily Tmax and Tmin during the study window was 106.0°F (102.0 – 109.0 °F IQR) and 84.0°F (79.0 – 88.0°F IQR), respectively. Figure 2 demonstrates the daily heatstroke survivors, deaths, and environmental Tmax and Tmin. Thirty-one (51.7%, 95% CI 38.4 – 64.8%) of the heatstroke presentations occurred in July, including seven (36.8%, 95% CI 16.3 – 61.6%) of the total study population deaths. The median July Tmax and Tmin were 106.5 °F (95% CI 102.3 – 111.0 °F) and 85.0°F (95% CI 80.3 – 89.0 °F), respectively. Heatstroke deaths represented 13.0% of all-cause ED deaths in July. In 2021 and 2022 there were 29 heat advisory dates. Nine (47.4%, 95% CI 24.5 – 71.1%) of the total heatstroke deaths occurred during heat advisory dates.

**Figure 3:**
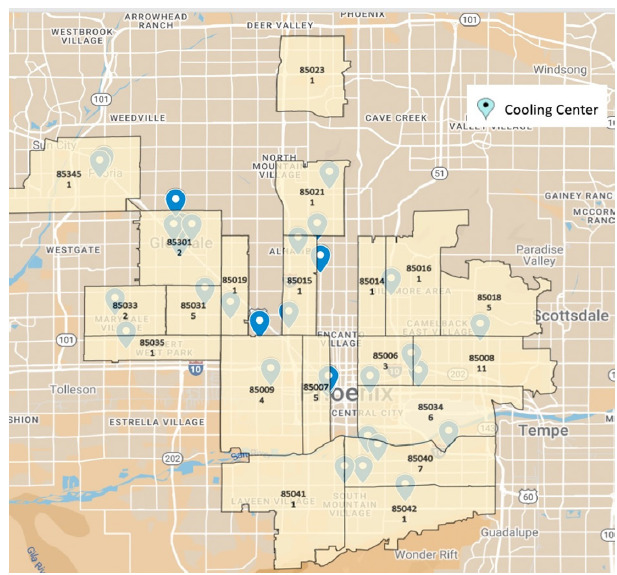
Heatstroke presentations and available cooling centers by Phoenix metropolitan-area ZIP code.

Heatstroke patients presented from 20 unique ZIP codes. Of these, 18 are in Phoenix and one each in the geographically adjacent cities of Glendale and Peoria. There were 23 and 31 publicly available cooling centers in the patient presentation ZIP codes in 2021 and 2022, respectively. The prehospital patient location and geographically available cooling resources are described by ZIP code in Figure 3.

## DISCUSSION

As a result of increasing environmental heat and exposure, the incidence and severity of heat illness and heatstroke are on the rise.^12^,^27^ In this study, a total of 60 presentations of heatstroke were identified at a single center from June 1–August 31, 2021, and June 1–August 31, 2022, in Phoenix, Arizona, with as many as four acute presentations and two deaths in a single day. In this population, heatstroke disproportionately impacted males (70.0%), persons experiencing homelessness (36.7%), and those with substance use disorder (53.3%). These results align with the 2022 MCDPH heat death report, which noted 424 heat-associated deaths, of which 81% of patients were male, 41.9% were experiencing homelessness, and 67% had associated substance use disorder.^12^ The acuity and severity of heat illness in the study population was significant with 85.3% requiring airway management, a 63.3% hospital admission rate, 81.6% of whom were admitted to the ICU, and 63.3% underwent cold water immersion. However, a substantial portion of the patients (68.3%) were ultimately discharged or transferred, 82.1% of whom were neurologically intact at the time.

As compared to prior literature, a significant portion of the heatstroke presentations in this study population did not adhere to the classical or exertional models.^19^ The “classic” heatstroke presentation commonly affects those with pre-existing health or socioeconomic conditions that result in the inability to appropriately avoid heat exposure.^19^,^28^,^29^ Alternatively, in the “exertional” presentation, patients are typically younger and exerting themselves beyond their ability to sufficiently dissipate heat.^19^ In this study, only nine (15.0%) presentations were clearly attributable to a classic cause of heatstroke. Most of the study population (47, 78.3%) presented after being found unresponsive in an outdoor urban environment, directly exposed to extreme heat. In this group, substance use disorder and homelessness appeared to play a significant role in their heat exposure and resultant illness. As compared to the remainder of the study cohort, this novel group was largely male (76.6%), experiencing homelessness (46.8%), and associated with substance use (methamphetamines 46.8%, fentanyl 29.8%) in those who were tested, which potentially underestimates the absolute prevalence in this population.

Prior literature has demonstrated an increased mortality in heat-exposed patients with concomitant substance use, particularly stimulants.^30^–^34^ Substance use likely contributes to the patient’s inability to appropriately avoid or dissipate heat, resulting in a heat-associated toxidrome, or heat poisoning. In the study population, the risk of heatstroke presentation and death was most specifically associated with methamphetamine and fentanyl use, potentially limiting the patient’s access to cooling resources, inhibiting the patient’s ability to respond to the normal biological heat-avoidance triggers, and prolonging heat exposure. The 2022 MCDPH statistics similarly identified the association of substance use in 67% of all heat deaths, including methamphetamines and fentanyl in 93% and 44.4% of patients, respectively.^12^ This novel trend poses unique challenges to the prevention and treatment of heat illness due to the combination of extreme weather exposure and substance use. In a 2014 study of Arizona cooling centers, the most frequent reasons for turning people away were safety concerns due to individual behaviors (56%) and intoxication (32%).^35^ The unique role that substance use plays in exacerbating heatstroke presentations in Phoenix, and likely beyond, cannot be underestimated and is deserving of further study to identify additional heat exposure prevention strategies in this population.

The treatment of heat illness in the prehospital and ED settings is logistically challenging. Cooling strategies are designed to induce rapid heat dissipation; these include the application of cold IV fluids and ice packs, evaporative cooling with fans, and cold water immersion.^1^,^19^ In the study population, 60.0% of the patients had documentation of prehospital-initiated cooling interventions. In the ED, cooling either continued or was initiated in 85% of encounters, with most undergoing cold water immersion (63.3%). While the overall non-immersion mortality rate was lower, cold water immersion was more commonly used in patients with a higher acuity (ESI 1 vs 2), intubation rate (78.9% vs 68.2%), rate of prehospital cardiac arrest (15.8% vs 7.7%), and core temperatures (median Tmax 42.0°C vs 40.7°C), which likely contributed to the higher mortality in this group. Despite this, cold water immersion therapy demonstrated a faster cooling rate (0.08°C per minute vs 0.04°C per minute) and shorter duration (median 35.0 minutes vs 60.5 minutes), cooling patients nearly twice as quickly as non-immersion therapy.^5^,^36^,^37^

Prehospital and ED cold water immersion therapy is often difficult to implement due to limited space, resources, and training.^14^ This is further complicated in patients presenting in cardiopulmonary arrest. In this study population, no heatstroke patients who presented to the ED in cardiopulmonary arrest survived to hospital admission. Of these patients 77.8% received no cooling interventions, potentially due to the challenging integration of cardiopulmonary resuscitation (CPR) with cooling interventions. However, prior studies have demonstrated simultaneous cold water immersion and ACLS, including CPR and defibrillation, to be safe and effective.^38^ The development of prehospital and ED cold water immersion therapy strategies, including the necessary resource allocation and training, which take into account cardiopulmonary arrest and the need for ACLS management, can potentially expedite cooling and improve outcomes in heatstroke patients with prehospital cardiopulmonary arrest, although they are challenging to implement.^38^,^39^

While the association between extreme environmental temperatures and the incidence of heatstroke and death is clear, a further understanding of the most relevant and predictable local weather metrics may help guide prevention strategies.^40^,^41^ In this study population, 36.8% of the patient deaths occurred in July, marked by the highest daily Tmax and Tmin. Similarly, in 2022 the MCDPH reported 58% of all heat deaths occurred in July and 25.2% occurred on days with an excessive heat warning.^12^ Predictive weather modeling is specific to the local climate. Warning systems use a variety of weather and heat metrics, such as daily mean, minimum, or maximum temperature, or composite indices of temperature and humidity, such as the heat index, to guide heat illness prevention strategies.^17^,^34^ Prior studies have also shown that heat-induced clinical presentations may counterintuitively begin to occur at moderate temperatures, below traditional advisory triggers.^8^,^40^ Heat advisory warning metrics and triggers may be challenging to standardize for both research and public health initiatives but should be adapted to local climates.^8^,^42^

A substantial portion of the study population was found to be undomiciled in an urban environment at the time of presentation. Similarly, Maricopa County heat death patients in 2022 were predominantly from outdoor and urban exposures (80%) and experiencing homelessness (41.9%).^12^ Prior literature has identified the highest heat mortality rates (0.3 per 100,000 population) in urban and metropolitan settings.^8^,^12^ Urban areas create “heat islands” due to the increased structural and impervious surface density, resulting in hotter daytime temperatures and limiting heat dissipation at night.^43^ The distribution and delivery of heat illness treatment and prevention resources to an urban and unhoused population can be challenging.^32^,^44^,^45^ “Heat vulnerability” maps, focused on urban areas and heat islands, may help predict areas at high risk for heat illness presentations, and guide the deployment of public health cooling resources.^46^

In this study population, the incidence and acuity of heatstroke presentation to a single urban system were significant and consistent with 2022 MCDPH data, which demonstrated 424 heatstroke deaths, a 25% increase in heat illness-associated deaths from 2021.^12^ For comparison, there were 660 motor vehicle deaths in Maricopa County in 2022.^47^ However, with timely cooling interventions, a substantial portion of the study patients were successfully discharged neurologically intact. These results demonstrate and support the need for significant and ongoing resource allocation to heat illness prevention and treatment. Additional investigation of this novel heatstroke presentation, including the associated risk of extreme weather, substance use, and outdoor and urban setting exposures is needed. Strategies should address the challenge of connecting public health and cooling resources to this high-risk patient population. Furthermore, the partnership of prehospital, public health, and emergency care systems is needed to optimize the prevention, early detection, and rapid treatment of heat illness.

## LIMITATIONS

During the development and implementation of this study several potential limitations were identified. The study population presented via EMS transport and represented a specific geographical sample of the Phoenix heatstroke cohort for the catchment area of the hospital system. The generalizability of the study population may be limited in less arid or rural settings. While this may not fully describe the entirety of the Phoenix metropolitan area, we do believe it is representative of a significant and important cohort of heatstroke presentations and is consistent with concurrent local public health reports.^12^

Additionally, certain patient demographic characteristics could not be fully obtained through retrospective chart extraction at the time of study development and analysis, limiting the ability to describe a selection of study patients in this analysis fully. These patients were included in the analysis as “unknown” where appropriate but may have led to underestimating certain study findings. Similarly, drug use or intoxication was defined by a positive urinary drug screen alone, but clinical features of intoxication could be consistently obtained through chart review.

Finally, the sample size was too small to be able to control for potential confounders; however, to our knowledge, this is the largest dataset of ED heatstroke patients reported. Future prospective and comprehensive studies in disparate climates and populations are warranted to better understand environmental and heat illness in this and dissimilar populations.

## CONCLUSION

In a single-center study of an urban population during the summers of 2021 and 2022, a significant number of heatstroke presentations and heat-related deaths were identified in Phoenix, Arizona. In this population, the extreme weather and association of substance use appeared to play a major role in precipitating heatstroke risk. However, a substantial portion of patients were successfully treated, often through cold water immersion, and discharged neurologically intact. Future studies evaluating the combined roles of public health, prehospital, and emergency care are necessary for the prevention and treatment of this environmentally mediated public health emergency.

## Supplementary Information



## Figures and Tables

**Table 1:** Demographics of patients who suffered heatstroke during the summers of 2021 and 2022.

Demographics	Adult heatstroke ED encounters (N = 60) (%, 95% CI)
Gender	
Male	42 (70.0%, 56.8 – 81.2%)
Female	14 (23.3%, 13.4 – 36.0%)
Unknown	4 (6.7%, 1.8 – 16.2%)
Age	
Years (mean)	52.7 (48.4 – 56.9)
Race	
Hispanic, Latino/a, or Spanish	12 (20.0%, 10.8 – 32.3%)
White	26 (43.3%, 30.6 – 56.8%)
Black	9 (15.0%, 7.1 – 26.6%)
American Indian or Alaska Native	5 (8.3%, 2.8 – 18.4%)
Other Asian	1 (1.7%, 0.04 – 8.9%)
Unknown	7 (11.7%, 4.8 – 22.6%)
Type of Insurance	
Medicaid (%)	37 (61.7%, 48.2 – 73.9%)
Medicare (%)	9 (15.0%, 7.1 – 26.6%)
Self-pay (%)	4 (6.7%, 1.8 – 16.2%)
Commercial (%)	2 (3.3%, 0.4 – 11.5%)
Unknown	8 (13.3%, 5.9 – 24.6%)
Domicile	
Domiciled	23 (38.3%, 26.1 – 51.8%)
Undomiciled	22 (36.7%, 24.6 – 50.1%)
Unknown	15 (25.0%, 14.7 – 37.9%)
ED arrival method	
Ground emergency medical services (%)	60 (100%)
Emergency Severity Index (ESI)	
ESI (median)	1 (IQR 1.0 – 2.0)

*ED*, emergency department; *IQR*, interquartile range

**Table 2:** Demographics and outcomes of patients who underwent cold water immersion cooling therapy.

Demographics	Cold water immersion encounters (N = 38) (%, 95% CI)
Age (Years, Mean)	50.2 (45.1–55.4)
Emergency Severity Index (Median, Interquartile Range)	1 (IQR 1.0–2.0)
Glasgow Coma Scale (Median, Interquartile Range)	3 (IQR 3.0–6.0)
Prehospital cooling	24 (63.2%, 46.0–78.2%)
Prehospital cardiopulmonary arrest	6 (15.8%, 6.0–31.3%)
Emergency department maximum temperature (median Fahrenheit)	42.0°F (41.3–42.3°F)
Prehospital intubation	2 (5.3%, 0.6–17.7%)
Emergency department intubation	28 (73.7%, 56.9–86.6%)
Outcome	
Death	11 (28.9%, 15.4–45.9%)
Discharge neurologically intact	20 (52.6%, 35.8–69.0%)
Discharge with neurological deficit	6 (15.8%, 6.0–31.3%)
Unknown disposition	1 (2.6%, 0.06–13.8%)

*IQR*, interquartile range

**Table 3:** Characteristics of patients with “unclassified” heat stroke.

Demographics	“Unclassified” heatstroke encounters (N = 47) (%, 95% CI)
Gender	
Male	36 (76.6%, 62.0 – 87.7%)
Female	8 (17.0%, 7.6– 30.8%)
Unknown	3 (6.4%, 1.3 – 17.5%)
Age (Mean, Years)	58.3 (50.9 – 65.7)
Housing	
Domiciled	12 (25.5%, 13.9 – 40.3%)
Homeless	22 (46.8%, 32.1 – 61.9%)
Unknown	13 (27.7%, 15.6 – 42.6%)
Temperature maximum (Median, Fahrenheit)	42.0°F (41.3 – 42.3)
Emergency department intubation	34 (72.3%, 57.4 – 84.4%)
Drug screen	
Methamphetamine	22 (46.8%, 32.1 – 61.9%)
Fentanyl	14 (29.8%, 17.3 – 44.9%)
Alcohol	4 (8.5%, 2.4 – 20.4%)
”Other”	16 (34.0%, 20.9 – 49.3%)
Negative	2 (4.3%, 0.52 – 14.5%)
Not obtained	18 (38.3%, 24.5 – 53.6%)
Prehospital naloxone	20 (42.6%, 28.3 – 57.8%)
Emergency department cooling method	
Cold water immersion	32 (68.1%, 52.9 – 80.9%)
Non-immersion cooling	9 (19.1%, 9.1 – 33.3%)
No cooling	6 (12.8%, 4.8 – 25.7%)
Outcome	
Death	15 (31.9%, 19.1 – 47.1%)
Discharge neurologically intact	26 (55.3%, 40.1 – 69.8%)
Discharge with neurological deficit	5 (10.6%. 3.5 – 23.1%)
Unknown disposition	1 (2.1%, 0.05 – 11.3%)
